# Patient Characteristics and Survival Outcomes of Non-Metastatic, Non-Clear Cell Renal Cell Carcinoma

**DOI:** 10.3389/fonc.2021.786307

**Published:** 2022-01-10

**Authors:** Josiah An, Vignesh T. Packiam, Adithya Chennamadhavuni, Jordan Richards, Jayanshu Jain, Sarah L. Mott, Rohan Garje

**Affiliations:** ^1^ Division of Hematology, Oncology, Blood & Marrow Transplantation, Department of Internal Medicine, University of Iowa Hospitals and Clinics, Iowa City, IA, United States; ^2^ Department of Urology, University of Iowa Hospitals and Clinics, Iowa City, IA, United States; ^3^ Department of Internal Medicine, University of Iowa Hospitals and Clinics, Iowa City, IA, United States; ^4^ Holden Comprehensive Cancer Center, University of Iowa Hospitals and Clinics, Iowa City, IA, United States

**Keywords:** renal cell carcinoma, papillary renal carcinoma, chromophobe carcinoma, sarcomatoid renal cell carcinoma, collecting duct carcinoma

## Abstract

**Background:**

Non-clear cell renal cell carcinoma (ccRCC) includes histologically and molecularly distinct subtypes such as papillary, chromophobe, collecting duct, and sarcomatoid RCC, with an incidence ranging from 20% to 25%. Oncologic outcomes and the role of adjuvant systemic therapy [vascular endothelial growth factor inhibitor (VEGFi) or immunotherapy] for non-ccRCC are not well-described.

**Objective:**

To assess the incidence and survival outcomes of non-ccRCC subtypes in comparison to ccRCC.

**Methods:**

The National Cancer Database was utilized to identify patients with non-metastatic RCC (T1–T4, N0–N1) between 2004 and 2015. The non-ccRCC cohort was further stratified by histologic subtype: papillary, chromophobe, sarcomatoid, and collecting duct RCC. Multivariable Cox regression models were used to compare overall survival (OS).

**Results:**

The 5-year OS for chromophobe, papillary, clear cell, collecting duct, and sarcomatoid RCC was 91%, 82%, 81%, 44%, and 40%, respectively. After adjusting for clinicopathologic and treatment characteristics, there was no significant difference in OS between papillary RCC and ccRCC (p = 0.17). Patients with collecting duct and sarcomatoid subtypes were at over two times increased risk of death compared to patients with clear cell (p < 0.01 and p < 0.01, respectively). Conversely, patients with chromophobe RCC were at 36% decreased risk of death compared to ccRCC (p < 0.01).

**Conclusions:**

This hospital-based analysis confirms that collecting duct and sarcomatoid histologic subtypes are uncommon and associated with poor survival after surgery when compared to the other RCC subtypes. Further studies are needed to evaluate the role of neoadjuvant and adjuvant systemic therapies in these subtypes to improve oncologic outcomes.

## Introduction

While the majority of renal cell carcinomas (RCCs) are clear cell RCC (ccRCC), non-ccRCCs comprise up to 25% of RCCs. Non-ccRCCs are further divided into histologic subtypes, including papillary (10%), chromophobe (5%), collecting duct (1%), and translocation RCC (<1%) (1). Sarcomatoid differentiation can be found in any subtype (2). A systematic review of 49 studies in 2015 found that systemic therapies for primarily metastatic RCC conferred poorer progression-free survival and overall survival (OS) in patients with non-ccRCC compared to those in patients with ccRCC (3). However, robust data regarding oncologic outcomes in patients with non-ccRCC, particularly with respect to neoadjuvant and adjuvant therapy, are more limited.

Currently, the standard treatment algorithm for localized or locoregional non-ccRCC is surgery followed by surveillance, mirroring management for ccRCC (4). While oncologic outcomes and the role of adjuvant therapy have not been well studied for localized non-ccRCC, there have been trials comparing systemic therapies in advanced non-ccRCC. Notably, the ESPN, ASPEN, and RECORD-3 trials compared sunitinib and everolimus (5–7). A meta-analysis found that sunitinib may be more effective than everolimus, but the difference in outcomes was not statistically significant (8). However, these trials were limited by not differentiating between histologic subtypes.

Recent molecular profiling has increasingly shown non-ccRCC to be a heterogenous disease (9). *MET* mutations are frequently present in papillary RCC, whereas mitochondrial alterations are frequently found in the chromophobe subtype (10, 11). The collecting duct subtype appears to be immunogenic, with upregulation of genes associated with T-cell activity along with lymphocytic infiltration on biopsy (12). Sarcomatoid RCCs may have increased frequency of mutations in *TP53*, *CKDN2A*, and *NF2* (13). These molecular differences suggest that different systemic therapies may be required for the various subtypes of non-ccRCC.

In light of the rarity and lack of robust data for localized non-ccRCC, we utilized a large hospital-based dataset to describe oncologic outcomes of papillary, chromophobe, collecting duct, and sarcomatoid subtypes of non-ccRCC in comparison to ccRCC.

## Methods

### Dataset

The National Cancer Database (NCDB) is an oncology database that represents more than 70% of newly diagnosed cancer cases nationwide. Data from more than 1,500 Commission on Cancer (CoC)-accredited programs contribute to patient demographics, treatments, and outcomes. These data represent more than 34 million historical records from 2004 to 2017.

The NCDB is a joint project of the Commission on Cancer of the American College of Surgeons and the American Cancer Society. The data used in the study were derived from a de-identified NCDB file. The American College of Surgeons and the CoC have not verified and are not responsible for the analytic or statistical methodology employed or the conclusions drawn from these data by the investigators.

### Ethics Statement

Institutional review board (IRB) approval and informed consent were not acquired according to the institutional guidelines.

### Patient Population

We identified patients with non-metastatic RCC (T1–T4, N0–N1) with histology including papillary, chromophobe, sarcomatoid, collecting duct, and ccRCC (primary site-specific codes: C649, C659) from 2004 to 2015 with age ≥18 years. We excluded patients with inadequate treatment and follow-up information, more than one cancer in a lifetime, and stage 0 or IV RCC. Patients were excluded if it was unknown whether they received radiation or chemotherapy or underwent surgery. In addition, patients without follow-up time beyond diagnosis or patients with unknown vital status were excluded.

### Clinicopathologic Features

We described and analyzed the following characteristics in the NCDB: facility type (non-academic, academic), facility location (central, mountain and Pacific, New England *vs*. Atlantic), number of cases at the facility (<15 cases per year, ≥15 cases per year), sex, age (<65 years, ≥65 years), race/ethnicity, primary payor, median income per zip code (<$48,000, ≥$48,000), area (urban/rural, metro), distance to hospital (<50 miles, ≥50 miles), Charlson–Deyo score (0–1, 2–3), histology (chromophobe, collecting duct, papillary, sarcomatoid, clear cell), NCDB analytic stage (stages I, II, III), surgery (no surgery, ablation/excision, partial, simple, radical), regional lymph node surgery (yes, no), radiation, and chemotherapy.

### Statistical Analysis

Baseline characteristics were summarized as frequency count and percentage. Cox regression models were used to assess the effect of patient and treatment factors with OS. Survival time was calculated from the date of diagnosis to death due to any cause; patients still alive were censored at last contact. To account for all discernible variables, including the possible dependency between patients seen at the same facility, a robust sandwich variance estimate was also used. In this model, patients are clustered within a facility; patients seen at the same facility will be more similarly treated than patients seen at different facilities. The failure times of patients within a cluster are then correlated, and the robust sandwich variance estimate adjusts for the intra-cluster correlation. Estimated effects are reported as hazard ratios (HRs) along with 95% confidence intervals. All tests were two-sided and assessed for significance at the 5% level using SAS v9.4 (SAS Institute, Cary, NC, USA). Because the patient, disease, and treatment characteristics under consideration are attempting to assess a latent variable process, it is necessary for the relationship between the covariates and OS to be evaluated jointly in the multivariable setting. In doing so, the unique contribution of each covariate to the hazard can be estimated while accounting for other confounding factors. Radiation is a component of some patients’ treatment regimen. Thus, it has been included in the multivariable model to ensure that the effect of each component of the treatment regimen is being uniquely estimated.

## Results

Overall, 220,170 RCC patients were included in our analysis. A total of 178,066 patients were found to have non-metastatic ccRCC and 42,104 with non-ccRCC. The majority of the non-ccRCC cohort was composed of papillary and chromophobe subtypes, 27,510 (65.3%) and 12,760 (30.3%), respectively. There were notable differences in the distribution of demographic characteristics and tumor-specific information (**Table 1**). Patients with papillary histology were more commonly male (72.6% male *vs*. 27.4% female), and chromophobe histology was more likely to present in patients below age 65 years compared to those above 65 years of age (65.2% *vs*. 34.8%).

**Table 1:** Patient demographics and treatment.

Covariate	Statistics	Level	Histology					
			Chromophobe	Clear Cell	Collecting Duct	Papillary	Sarcomatoid	Overall
			N = 12,760	N = 178,066	N = 273	N = 27,510	N = 1,561	N = 220,170
Facility Type	N (Col %)	Academic	5,296 (46.2)	66,253 (39.5)	104 (41.4)	12,212 (46.3)	644 (42.6)	84,509 (40.8)
	N (Col %)	Non-academic	6,155 (53.8)	101,498 (60.5)	147 (58.6)	14,174 (53.7)	867 (57.4)	122,841 (59.2)
	N (Col %)	Missing	1,309	10,315	22	1,124	50	12,820
Facility Location	N (Col %)	Central	4,456 (38.9)	76,445 (45.6)	94 (37.5)	11,128 (42.2)	693 (45.9)	92,816 (44.8)
	N (Col %)	Mountain and Pacific	1,818 (15.9)	24,532 (14.6)	47 (18.7)	2,954 (11.2)	196 (13.0)	29,547 (14.2)
	N (Col %)	New England and Atlantic	5,177 (45.2)	66,774 (39.8)	110 (43.8)	12,304 (46.6)	622 (41.2)	84,987 (41.0)
	N (Col %)	Missing	1,309	10,315	22	1,124	50	12,820
Cases for Year of Diagnosis	N (Col %)	<15 cases	1,663 (13.0)	30,702 (17.2)	54 (19.8)	3,628 (13.2)	270 (17.3)	36,317 (16.5)
	N (Col %)	15+ cases	11,097 (87.0)	147,364 (82.8)	219 (80.2)	23,882 (86.8)	1,291 (82.7)	183,853 (83.5)
Sex	N (Col %)	Male	6,813 (53.4)	104,834 (58.9)	166 (60.8)	19,983 (72.6)	977 (62.6)	132,773 (60.3)
	N (Col %)	Female	5,947 (46.6)	73,232 (41.1)	107 (39.2)	7,527 (27.4)	584 (37.4)	87,397 (39.7)
Age	N (Col %)	<65 years	8,319 (65.2)	107,568 (60.4)	153 (56.0)	16,014 (58.2)	885 (56.7)	132,939 (60.4)
	N (Col %)	65+ years	4,441 (34.8)	70,498 (39.6)	120 (44.0)	11,496 (41.8)	676 (43.3)	87,231 (39.6)
Race/Ethnicity	N (Col %)	Black	1,578 (12.5)	15,543 (8.8)	65 (24.3)	7,142 (26.3)	156 (10.1)	24,484 (11.2)
	N (Col %)	Hispanic	935 (7.4)	13,433 (7.6)	8 (3.0)	935 (3.4)	108 (7.0)	15,419 (7.1)
	N (Col %)	Other	374 (3.0)	5,539 (3.1)	9 (3.4)	510 (1.9)	49 (3.2)	6,481 (3.0)
	N (Col %)	White	9,721 (77.1)	141,690 (80.4)	186 (69.4)	18,614 (68.4)	1,235 (79.8)	171,446 (78.7)
	N (Col %)	Missing	152	1,861	5	309	13	2,340
Primary Payor	N (Col %)	Not insured	339 (2.7)	6,439 (3.7)	8 (3.0)	721 (2.7)	63 (4.2)	7,570 (3.5)
	N (Col %)	Private	7,089 (56.9)	84,749 (48.7)	114 (42.5)	12,243 (45.4)	723 (47.7)	104,918 (48.8)
	N (Col %)	Public	5,041 (40.4)	82,786 (47.6)	146 (54.5)	13,991 (51.9)	731 (48.2)	102,695 (47.7)
	N (Col %)	Missing	291	4,092	5	555	44	4,987
Median Income Quartiles 2008–2012	N (Col %)	<$48,000	4,528 (35.6)	75,235 (42.4)	129 (47.6)	11,820 (43.1)	667 (42.8)	92,379 (42.1)
	N (Col %)	$48,000+	8,190 (64.4)	102,041 (57.6)	142 (52.4)	15,579 (56.9)	892 (57.2)	126,844 (57.9)
	N (Col %)	Missing						947
Area	N (Col %)	Metro	10,708 (86.2)	141,625 (81.6)	227 (85.3)	22,812 (85.1)	1,223 (80.5)	176,595 (82.3)
	N (Col %)	Urban/Rural	1,709 (13.8)	31,986 (18.4)	39 (14.7)	3,982 (14.9)	297 (19.5)	38,013 (17.7)
	N (Col %)	Missing	343	4,455	7	716	41	5,562
Distance to hospital	N (Col %)	<50 miles	10,781 (84.7)	149,383 (84.2)	240 (88.6)	23,372 (85.2)	1,280 (82.1)	185,056 (84.3)
	N (Col %)	50+ miles	1,948 (15.3)	28,080 (15.8)	31 (11.4)	4,049 (14.8)	279 (17.9)	34,387 (15.7)
	N (Col %)	Missing	31	603	2	89	2	727
Charlson–Deyo Score	N (Col %)	0–1	12,058 (94.5)	161,911 (90.9)	241 (88.3)	24,875 (90.4)	1,447 (92.7)	200,532 (91.1)
	N (Col %)	2–3	702 (5.5)	16,155 (9.1)	32 (11.7)	2,635 (9.6)	114 (7.3)	19,638 (8.9)
Grade	N (Col %)	Well differentiated	653 (8.2)	20,534 (14.5)	16 (7.2)	2,793 (13.1)	35 (2.9)	24,031 (14.0)
	N (Col %)	Moderately differentiated	4,356 (54.6)	78,211 (55.3)	42 (19.0)	11,595 (54.5)	111 (9.2)	94,315 (54.8)
	N (Col %)	Poorly or Undifferentiated	2,972 (37.2)	42,604 (30.1)	163 (73.8)	6,905 (32.4)	1,058 (87.9)	53,702 (31.2)
	N (Col %)	Missing	4779	36,717	52	6,217	357	48,122
Stage	N (Col %)	Stage I	8,760 (68.7)	130,407 (73.2)	112 (41.0)	21,857 (79.5)	429 (27.5)	161,565 (73.4)
	N (Col %)	Stage II	2,442 (19.1)	19,171 (10.8)	27 (9.9)	3,070 (11.2)	271 (17.4)	24,981 (11.3)
	N (Col %)	Stage III	1,558 (12.2)	28,488 (16.0)	134 (49.1)	2,583 (9.4)	861 (55.2)	33,624 (15.3)
Surgery	N (Col %)	None	167 (1.3)	10,245 (5.8)	8 (2.9)	674 (2.5)	45 (2.9)	11,139 (5.1)
	N (Col %)	Ablation/Excision	401 (3.1)	10,816 (6.1)	2 (0.7)	1,884 (6.8)	23 (1.5)	13,126 (6.0)
	N (Col %)	Partial	4,654 (36.5)	53,142 (29.8)	35 (12.8)	11,904 (43.3)	157 (10.1)	69,892 (31.7)
	N (Col %)	Simple	1,390 (10.9)	19,396 (10.9)	56 (20.5)	2,723 (9.9)	217 (13.9)	23,782 (10.8)
	N (Col %)	Radical	6,148 (48.2)	84,467 (47.4)	172 (63.0)	10,325 (37.5)	1,119 (71.7)	102,231 (46.4)
Regional Lymph Node Surgery	N (Col %)	No	11,233 (88.8)	159,587 (90.4)	192 (71.1)	25,113 (92.0)	999 (64.9)	197,124 (90.3)
	N (Col %)	Yes	1,417 (11.2)	16,866 (9.6)	78 (28.9)	2,176 (8.0)	541 (35.1)	21,078 (9.7)
	N (Col %)	Missing	110	1,613	3	221	21	1,968
Radiation	N (Col %)	No	12,746 (99.9)	177,492 (99.7)	265 (97.1)	27,462 (99.8)	1,534 (98.3)	219,499 (99.7)
	N (Col %)	Yes	14 (0.1)	574 (0.3)	8 (2.9)	48 (0.2)	27 (1.7)	671 (0.3)
Chemotherapy	N (Col %)	No	12,655 (99.2)	175,643 (98.6)	240 (87.9)	27,250 (99.1)	1,403 (89.9)	217,191 (98.6)
	N (Col %)	Yes	105 (0.8)	2,423 (1.4)	33 (12.1)	260 (0.9)	158 (10.1)	2,979 (1.4)

At the time of diagnosis or treatment, there were 73.8% collecting duct and 87.9% sarcomatoid RCC patients with poorly or undifferentiated histology. Conversely, only 37.2%, 30.1%, and 32.4% patients with chromophobe, clear cell, and papillary RCC had poorly or undifferentiated histology, respectively. Similarly, 49.1% and 55.2% of patients with collecting duct and sarcomatoid histology had stage III disease. While 12.2%, 16.0%, and 9.4% patients with chromophobe, clear cell, and papillary RCC had stage III disease, respectively.

A higher proportion of patients with collecting duct (63.0%) and sarcomatoid (71.7%) histology underwent radical surgery compared to patients with chromophobe, clear cell, and papillary histology. Similarly, regional lymph node surgery was performed in 28.9% and 35.1% patients with collecting duct and sarcomatoid histology. Regional lymph node surgery was less common in the remaining subtypes. In terms of radiation therapy, most patients with RCC did not receive radiation treatment. A higher proportion of patients with collecting duct (12.1%) and sarcomatoid (10.1%) RCC received chemotherapy compared to other histologies.

### Survival Outcomes

Median follow-up for all histologies was 48.5 months. Chromophobe RCC was associated with favorable OS, with a 5-year OS of 91%. Clear cell and papillary subtypes had similar 5-year OS of 82% and 81%, respectively (**Table 2**). Collecting duct and sarcomatoid RCCs were associated with worse outcome, each having 44% and 40% 5-year OS, respectively.

**Table 2:** The 5-year OS of non-ccRCC compared to ccRCC.

Variable	Level	5-Year OS (95% CI)
Histology	Clear Cell	81% (80%–81%)
	Chromophobe	91% (90%–92%)
	Collecting Duct	44% (37%–50%)
	Papillary	82% (82%–83%)
	Sarcomatoid	40% (37%–43%)

ccRCC, clear cell renal cell carcinoma; OS, overall survival.

### Multivariable Analysis

Multivariable analysis included 6,723 chromophobe, 188 collecting duct, 19,086 papillary, and 1,083 sarcomatoid RCC with stage I–III RCC (**Table 3**). Age ≥65 years was associated with worse survival compared to patients aged <65 years (HR = 1.75, 95% CI: 1.69–1.82). Male sex (HR = 1.16, 95% CI: 1.13–1.19) was associated with worse outcome compared to female sex. Black race (HR = 1.11, 95% CI: 1.06–1.16) was associated with lower OS compared to White patients, while Hispanic (HR = 0.76, 95% CI 0.72–0.81) and Other (HR = 0.82, 95% CI 0.75–0.90) were associated with greater survival. Facilities with <15 cases per year was associated with worse outcome (HR = 1.07, 95% CI: 1.03–1.10) compared to facilities that treat ≥15 patients per year.

**Table 3:** Multivariable results for patient demographics and treatment.

Covariate	Level	N	Overall Survival			
			Hazard Ratio	95% CI		p-value
Facility Type	Non-academic	91,200	1.00	0.96	1.04	0.98
	Academic	60,056	Ref	-	-	
Facility Location	Central	68,701	1.06	1.02	1.10	0.03
	Mountain and Pacific	21,949	1.03	0.98	1.09	
	New England and Atlantic	60,606	Ref	-	-	
Cases for Year of Diagnosis	<15 cases	24,784	1.07	1.03	1.10	<0.01
	15+ cases	126,472	Ref	-	-	
Sex	Male	91,605	1.16	1.13	1.19	<0.01
	Female	59,651	Ref	-	-	
Age	65+ years	60,644	1.75	1.69	1.82	<0.01
	<65 years	90,612	Ref	-	-	
Race/Ethnicity	Black	15,920	1.11	1.06	1.16	<0.01
	Hispanic	10,083	0.76	0.72	0.81	
	Other	4,365	0.82	0.75	0.90	
	White	120,888	Ref	-	-	
Primary Payor	Not insured	4,859	1.42	1.31	1.54	<0.01
	Public	71,712	1.68	1.62	1.75	
	Private	74,685	Ref	-	-	
Median Income Quartiles 2008–2012	<$48,000	63,785	1.18	1.15	1.22	<0.01
	$48,000+	87,471	Ref	-	-	
Area	Urban/Rural	27,507	0.98	0.94	1.01	0.21
	Metro	123,749	Ref	-	-	
Distance to hospital	50+ miles	23,351	1.04	1.00	1.08	0.08
	<50 miles	127,905	Ref	-	-	
Charlson–Deyo Score	2–3	13,818	1.99	1.92	2.06	<0.01
	0–1	137,438	Ref	-	-	
Grade	Well differentiated	21,089	0.71	0.69	0.73	<0.01
	Moderately differentiated	82,571	0.70	0.67	0.73	
	Poorly or Undifferentiated	47,596	Ref	-	-	
Histology	Chromophobe	6,723	0.64	0.59	0.68	<0.01
	Collecting Duct	188	2.26	1.82	2.82	
	Papillary	19,086	1.03	0.99	1.07	
	Sarcomatoid	1,083	2.60	2.33	2.91	
	Clear Cell	124,176	Ref	-	-	
NCDB Analytic Stage Group	Stage II	17,695	1.35	1.31	1.40	<0.01
	Stage III	24,540	1.94	1.88	2.00	
	Stage I	109,021	Ref	-	-	
Surgery	None	1,787	2.92	2.68	3.18	<0.01
	Ablation/Excision	5,660	1.19	1.12	1.27	
	Partial	49,597	0.61	0.58	0.63	
	Simple	16,100	1.00	0.96	1.04	
	Radical	78,112	Ref	-	-	
Regional Lymph Node Surgery	Yes	15,465	1.24	1.19	1.28	<0.01
	No	135,791	Ref	-	-	
Radiation	Yes	350	1.90	1.58	2.29	<0.01
	No	150,906	Ref	-	-	
Chemotherapy	Yes	1,942	1.79	1.64	1.95	<0.01
	No	149,314	Ref	-	-	

Well-differentiated (HR = 0.71, 95% CI: 0.69–0.73) and moderately differentiated (HR = 0.70, 95% CI: 0.67–0.73) histologies were associated with better outcome. Compared to stage I analytic stage group, stages II (HR = 1.35, 95% CI: 1.31–1.40) and III (HR = 1.94, 95% CI: 1.88–2.00) were associated with worse outcome. In terms of surgical treatment, compared to radical resection, no surgical treatment (HR = 2.92, 95% CI: 2.68–3.18) and ablation/excision (HR = 1.19, 95% CI: 1.12–1.27) were associated with increased adverse outcome. Interestingly, partial resection was associated with improved OS (HR = 0.61, 95% CI: 0.58–0.63) compared to radical resection, while OS with simple resection was not significantly different from radical resection. Regional lymph node surgery was associated with worse survival outcome (HR = 1.24, 95% CI: 1.19–1.28) compared to no regional lymph node surgery. Radiation treatment (HR = 1.90, 95% CI: 1.58–2.29) was associated with adverse outcome, and patients who received chemotherapy had decreased survival (HR = 1.79, 95% CI: 1.64–1.95). Relative to clear cell, collecting duct (HR = 2.26, 95% CI: 1.82–2.82) and sarcomatoid (HR = 2.60, 95% CI: 2.33–2.91) were associated with poorer OS, chromophobe (HR = 0.64, 95% CI: 0.59–0.68) was associated with better OS, and papillary (HR = 1.03, 95% CI: 0.99–1.07) was not significantly different.

## Discussion

We analyzed a large hospital-based dataset to demonstrate several key findings regarding non-ccRCC. Our results demonstrate that 1) patients with collecting duct and sarcomatoid RCC present with higher stage and have significantly inferior oncologic outcomes relative to other subtypes and 2) patients who received radiation or chemotherapy had worse OS, although they are likely some of the sickest patients with other unmeasured risk factors (genetic mutations) for poor outcomes for any non-ccRCC subtype. These findings highlight the unmet need for more effective therapies for non-ccRCC.

Our findings are consistent with the existing literature regarding presenting characteristics of non-ccRCC. As demonstrated in prior studies, patients with the collecting duct and sarcomatoid subtypes presented more commonly with clinical T3+ and metastatic than regional disease (14, 15). Additionally, case volume was associated with outcomes, as patients treated at facilities that treat <15 cases per year experienced worse survival in our analysis. Black race was associated with both worse presentation and worse outcomes relative to others. Nearly a quarter of collecting duct RCC was observed in the Black race and was found to have higher grade and stage. Also, carrying no health insurance or public insurance was associated with worse survival, and earning <$48,000 per residential zip code was associated with worse outcomes.

**Figure 1:**
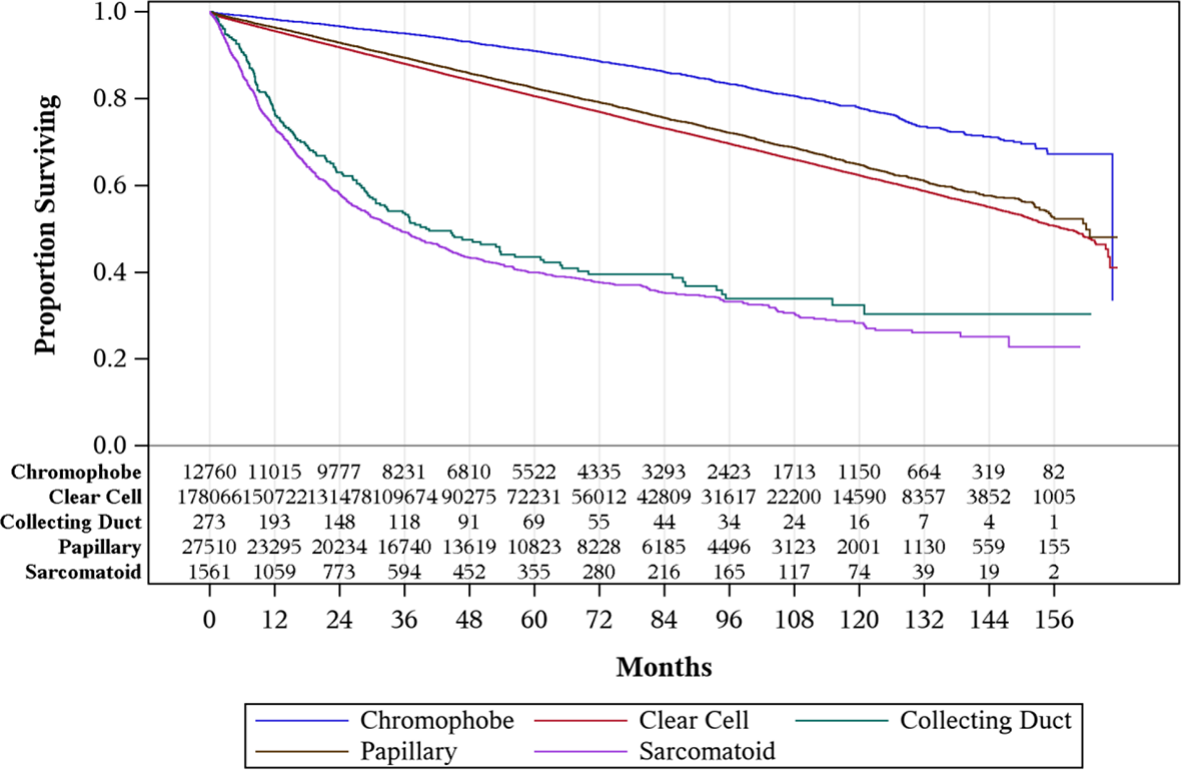
Overall survival by histology for stages I–III renal cell carcinoma (RCC).

With respect to oncologic outcomes, our findings also confirm that collecting duct and sarcomatoid histologies are associated with worse survival compared to other types of RCC, with over two times increased risk of death compared to patients with ccRCC. Conversely, patients with chromophobe were at 36% decreased risk of death compared to the clear cell, which is consistent with prior literature (15). OS for papillary RCC was not found to be significantly different from RCC (**Figure 1**). Other findings may have been influenced by the small sample size and uncontrolled selection bias: 1) regional lymphadenectomy was associated with worse outcomes, 2) adjuvant radiation was not associated with improved survival, and 3) adjuvant chemotherapy was not associated with improved survival. Vascular endothelial growth factor (VEGF), mammalian target of rapamycin (mTOR), and immune checkpoint inhibitors have shown significant clinical benefit in metastatic RCC (16). These agents are being evaluated in the adjuvant setting for non-metastatic RCC. For patients with high-risk stage III ccRCC, adjuvant sunitinib was approved by the Food and Drug Administration (FDA) in 2017 based on the S-TRAC trial, which showed improved disease-free survival (DFS) relative to placebo (17). However, due to lack of OS benefit and concerns with toxicity, adjuvant sunitinib is infrequently administered in practice. In the ASSURE clinical trial, which assessed patients with resected RCC, of whom 21% had non-ccRCC, adjuvant sunitinib or sorafenib did not show DFS benefit in comparison to placebo (18). Due to these studies, adjuvant sunitinib for stage I–III non-ccRCC is not recommended.

Currently, the role of adjuvant immune checkpoint inhibitors is unknown. In the metastatic setting, the combination of nivolumab and ipilimumab has shown better progression-free survival and OS in comparison to sunitinib in patients with metastatic sarcomatoid RCC (19). Currently, there are multiple ongoing phase III clinical trials evaluating the role of immune checkpoint inhibitors in the adjuvant setting of RCC with or without sarcomatoid features (20). In the adjuvant study evaluating the efficacy of nivolumab, any RCC histologic subtype is allowed.

Our study has several limitations. Due to the rarity of collecting duct and sarcomatoid subtypes, despite our inclusion of more than 220,000 patients with stage I–III RCC, there were only 273 patients with collecting duct and 1,561 patients for sarcomatoid histology, and the number of patients for both subtypes decreased even further with multivariate analysis, as patients without matching variables were excluded from the multivariate analysis. Furthermore, the NCDB dataset is limited by additional unknown confounding variables and missing data. NCDB does not collect specific granular treatment-related information regarding systemic therapy such as chemotherapy, VEGF, mTOR inhibitors, or immunotherapy types, and hence, it was not feasible to derive more specific treatment-associated outcomes. Limited economic factors included in the NCDB are also adjusted for in the multivariable model. While the patient’s income is not collected by NCDB, the median income for the ZIP code the patient lives in is included. Additionally, primary payor, area, and distance to hospital can be considered proxies for access to care. After adjusting for these available metrics, black race was still significantly associated with increased risk of death relative to white race. However, the statistically significant result may not have practical implications given the smaller in magnitude HR evidenced (HR = 1.11). Lastly, renal medullary and translocation RCC were not available as distinct histologies on NCDB; therefore, they were not included in this study.

## Conclusion

Collecting duct and sarcomatoid histologies were uncommon but were more likely to present with higher stage and have worse survival when compared to other RCC subtypes. Consistent with the current standard of care, surgical treatment for stage I–III RCC showed the greatest survival benefit compared to those without surgery or ablation procedures only. Further studies are needed to evaluate effective adjuvant treatment for non-ccRCC, particularly to improve outcomes for the collecting duct and sarcomatoid subtypes due to significantly worse survival compared to other subtypes.

## Data Availability Statement

The original contributions presented in the study are included in the article/supplementary material. Further inquiries can be directed to the corresponding author.

## Author Contributions

JA: Conception and design, collection and assembly of data, data analysis and interpretation, article writing, final approval of article. VP: Conception and design, collection and assembly of data, data analysis and interpretation, article writing, final approval of article. AC: Conception and design, article writing, final approval of article. JR: Data analysis and interpretation, article writing, final approval of article. JJ: Data analysis and interpretation, article writing, final approval of article. SM: Collection and assembly of data, data analysis and interpretation, article writing, final approval of article. RG: Conception and design, collection and assembly of data, data analysis and interpretation, article writing, final approval of article. All authors (a) have made substantial contributions to the work including conception, performance, or interpretation of data and (b) writing the article, (c) approved the final version to be published, and (d) agreed to be accountable for the accuracy and integrity of the work.

## Funding

Department funding by the Division of Hematology and Oncology at the University of Iowa Hospitals and Clinics.

## Conflict of Interest

RG received research funding from Endocyte/Advanced Accelerator Applications and Pfizer. VP has done consulting for Cold Genesys and Guidepoint.

The remaining authors declare that the research was conducted in the absence of any commercial or financial relationships that could be construed as a potential conflict of interest.

## Publisher’s Note

All claims expressed in this article are solely those of the authors and do not necessarily represent those of their affiliated organizations, or those of the publisher, the editors and the reviewers. Any product that may be evaluated in this article, or claim that may be made by its manufacturer, is not guaranteed or endorsed by the publisher.
